# OsbZIP60 Positively Regulates Salt-Stress Tolerance in Rice

**DOI:** 10.3390/ijms27188143

**Published:** 2026-09-12

**Authors:** Liqun Tang, Honghuan Fan, Junmin Wang, Kaizhen Zhong, Kunquan Liu, Mingli Han, Jian Song

**Affiliations:** 1 Institute of Crops and Nuclear Technology Utilization, Zhejiang Academy of Agricultural Sciences, Hangzhou 310021, China; liquntang2013@126.com (L.T.); xixi615@163.com (H.F.); wangjm917@sina.com (J.W.); zkz0804@163.com (K.Z.); liukq@zaas.ac.cn (K.L.); 2 Huzhou Academy of Agricultural Sciences, Huzhou 313000, China; winzju@163.com

**Keywords:** rice (*Oryza sativa* L.), salt tolerance, *OsbZIP60*, antioxidant defense, Na^+^/K^+^ ion homeostasis

## Abstract

Soil salinity is a major abiotic stress limiting rice growth and grain productivity worldwide. Basic leucine zipper (bZIP) transcription factors serve as central regulators of plant environmental stress responses, yet the biological function and molecular regulatory mechanism of rice *OsbZIP60* (*LOC_Os07g44950*) underlying salinity tolerance remain largely uncharacterized. In this study, we systematically characterized the salt-stress regulatory function of *OsbZIP60* in rice. Tissue expression profiling revealed that *OsbZIP60* was ubiquitously transcribed across all examined rice tissues, and its encoded protein predominantly localizes to the cell nucleus. Transcript abundance of *OsbZIP60* was significantly induced by salt, the osmotic phase, abscisic acid (ABA), and oxidative stress signals. Phenotypic assays demonstrated that overexpression of *OsbZIP60* substantially enhanced rice salt tolerance, whereas the *bzip60* knockout mutant exhibited aggravated salt hypersensitivity. Physiological quantification revealed that *OsbZIP60* promoted the accumulation of osmoprotectants, alleviated salt-triggered oxidative damage, and elevated the activities of core antioxidant enzymes under saline conditions. Moreover, OsbZIP60 maintained intracellular Na^+^/K^+^ homeostasis and positively modulated the transcript levels of a series of salt-responsive downstream genes. Collectively, our results demonstrate that *OsbZIP60* acts as a positive regulatory hub that coordinates osmotic adjustment, antioxidant defense, and ion balance to confer salt tolerance in rice, providing a promising genetic target for molecular breeding of salt-tolerant rice varieties.

## 1. Introduction

Soil salinity represents one of the most severe abiotic stresses that substantially constrain global agricultural productivity [1,2]. Excess soluble salts in soil induce multiple detrimental effects in crop plants, including cellular osmotic imbalance, toxic-ion accumulation, and secondary oxidative damage [3,4]. These physiological disturbances collectively impair seed germination, vegetative growth, photosynthetic efficiency, and ultimately grain yield [5,6]. Rice (*Oryza sativa* L.), a staple food crop feeding more than half of the global population, is highly sensitive to salt-stress, particularly during seed germination, seedling establishment, and reproductive developmental stages [7]. Therefore, identifying salt-responsive genes and elucidating their underlying regulatory mechanisms are essential for the genetic improvement of salt-tolerant rice cultivars.

Plant salt tolerance is governed by complex and coordinated physiological and molecular networks. Maintenance of intracellular Na^+^/K^+^ homeostasis constitutes a core adaptive strategy for rice to cope with salinity stress [8,9,10,11,12]. The sodium transporter *OsHKT1;5* plays an essential role in restricting excessive Na^+^ accumulation in rice shoots, and its natural genetic variation is tightly associated with rice salt-tolerance phenotypes [13,14]. Accumulating multi-omics analyses and genome-wide association studies (GWAS) have identified a series of key regulators involved in rice salt tolerance, including *STG5/qSTS5*, *OsWRKY53* and *RST1*, as well as multiple quantitative trait loci (QTLs) and candidate genes involved in Na^+^/K^+^ balance, nitrogen metabolism, and stress-related transcriptional regulation [15,16,17,18]. Beyond ion homeostasis, osmotic adjustment mediated by compatible osmolytes (e.g., soluble sugars and proline) is indispensable for sustaining cellular water potential and membrane stability under salt-stress [19,20].

Salt-stress also triggers rapid burst and accumulation of reactive oxygen species (ROS), such as superoxide anion and hydrogen peroxide (H_2_O_2_) [21,22,23,24]. At low physiological concentrations, ROS function as important signaling molecules in plant stress responses; however, excessive ROS accumulation provokes severe lipid peroxidation, irreversible membrane damage, and photosynthetic dysfunction [25,26]. To alleviate oxidative injury and maintain intracellular redox homeostasis, plants activate integrated enzymatic and non-enzymatic antioxidant defense systems. Core ROS-scavenging enzymes, including superoxide dismutase (SOD), peroxidase (POD), catalase (CAT), and ascorbate peroxidase (APX), are primarily responsible for eliminating excess ROS and conferring salt-stress tolerance [27,28]. Consistent with this mechanism, numerous studies have demonstrated that salt-tolerant rice genotypes and transgenic lines exhibit enhanced antioxidant capacity and reduced malondialdehyde (MDA) accumulation under salinity conditions [29,30,31].

Transcription factors serve as central regulatory hubs connecting upstream stress perception with downstream defense-response activation [32,33,34]. Multiple transcription-factor families, including bZIP, AP2/ERF, NAC, MYB, WRKY, MADS, and GRF, have been widely documented to participate in plant salt-stress adaptation [35,36,37,38,39,40,41]. In rice, *OsSAE1* positively regulates salt tolerance and seed germination by repressing *OsABI5*-mediated ABA signaling [7]. *OsMADS27* modulates nitrate-dependent salt tolerance via regulating ion homeostasis and antioxidant-gene expression [19]. The *miR396b-GRF6* regulatory module enhances salt resistance by reducing H_2_O_2_ accumulation and activating ROS-scavenging pathways [27]. These findings highlight the crucial role of transcriptional regulatory networks in rice salt-stress adaptation.

Among diverse transcription-factors, the bZIP transcription factor family has attracted extensive research attention, as its members widely participate in ABA signal transduction and abiotic-stress responses in plants. *OsbZIP23* is a well-characterized positive regulator of ABA signaling and salt tolerance in rice, and the *OsbZIP23–UGT2* module improves salt resistance by activating downstream stress-responsive pathways [42,43]. Other bZIP members, such as *OsABI5* and *OsbZIP71*, also modulate seed germination, osmotic adaptation, and salt-stress tolerance [44,45,46]. The bZIP60 subfamily has been functionally characterized in several plant species. In *Arabidopsis thaliana*, AtbZIP60 is a well-known master regulator of the endoplasmic reticulum (ER) unfolded-protein response [47,48]. In pyrethrum (*Tanacetum cinerariifolium*), TcbZIP60 responds to ABA and jasmonic acid and positively modulates pyrethrin biosynthesis [49]. Overexpression of transcription factor *OsbZIP60* affects heat and drought tolerance in rice [50]. Nevertheless, the biological role of rice *OsbZIP60* (*LOC_Os07g44950*) remains largely uninvestigated. Preliminary transcriptome performed in our laboratory identified that *OsbZIP60* transcript abundance was strongly induced under high-salinity conditions, prompting us to dissect its function in rice salt-stress responses.

In this study, we systematically characterized the salt-stress-regulatory function of *OsbZIP60* in rice via tissue-expression profiling, subcellular-localization observation, phenotypic identification of transgenic materials, physiological-index quantification, and stress-responsive gene expression detection. The results revealed that *OsbZIP60* expression is significantly induced by NaCl, PEG-simulated osmotic stress, ABA, and H_2_O_2_. Functional verification demonstrated that *OsbZIP60* overexpression markedly enhances rice salt tolerance, while *bzip60* knockout mutant compromises stress resistance. Collectively, our results indicate that *OsbZIP60* acts as a positive regulator of rice salinity tolerance by coordinately modulating ion homeostasis, osmolyte accumulation, antioxidant defense, and downstream stress-responsive gene expression.

## 2. Results

### 2.1. OsbZIP60 Is Expressed in Multiple Rice Tissues

**Figure 1:**
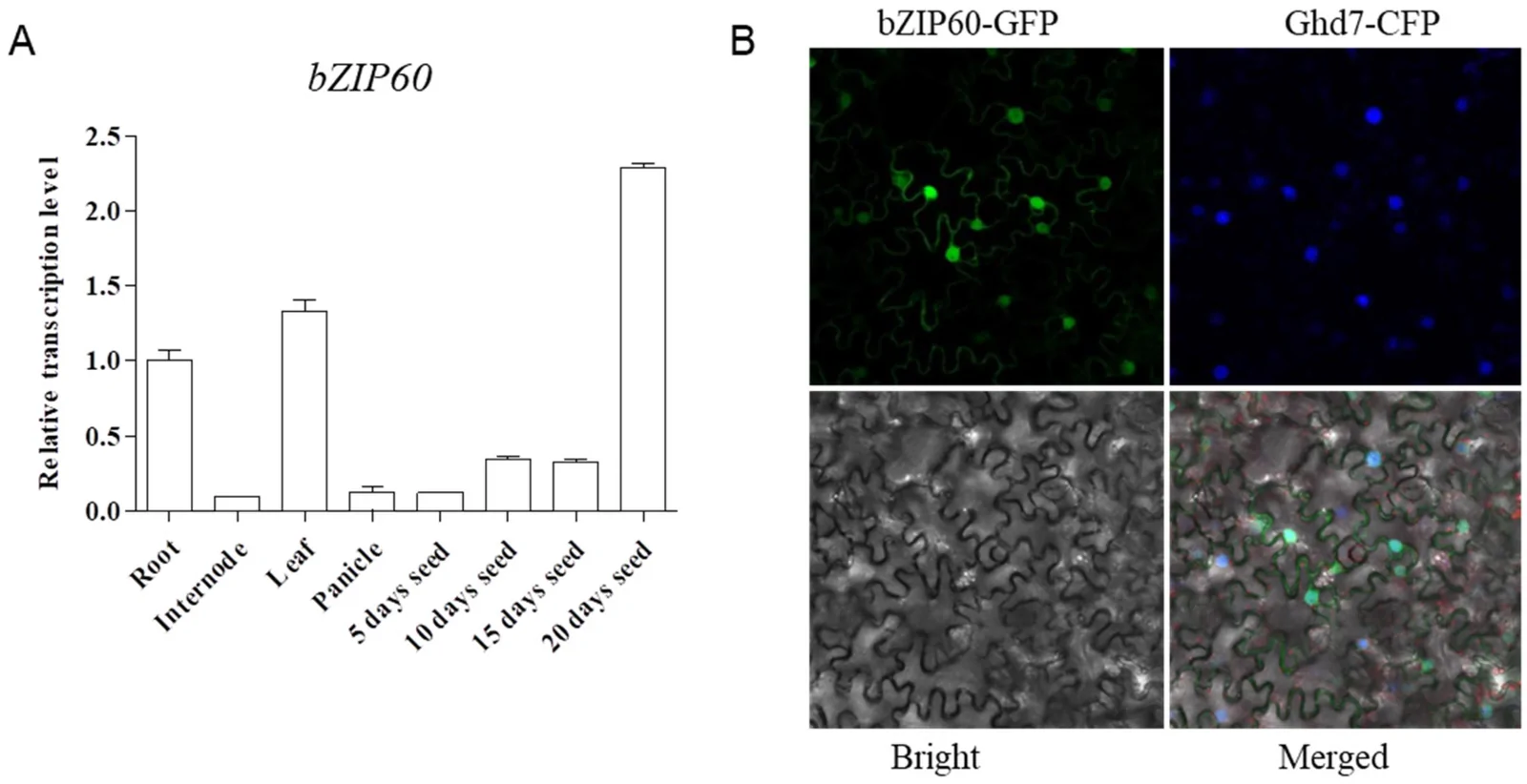
Expression pattern and subcellular localization of OsbZIP60. (**A**) Relative transcript levels of *OsbZIP60* in different rice tissues, including root, internode, leaf, panicle and developing seeds at 5, 10, 15 and 20 days after flowering. (**B**) Subcellular localization of OsbZIP60-GFP. OsbZIP60-GFP fluorescence was visualized together with the nuclear marker Ghd7-CFP. Bright-field and merged fluorescence images are presented. Error bars represent standard deviation (SD) of biological replicates; only upper-direction error bars are shown in the plot. Different lowercase letters indicate significant differences at *p* < 0.05 (*n* = 3).

To characterize the basal expression pattern of *OsbZIP60*, we quantified its relative transcript levels across different rice tissues. The transcriptional signal of *OsbZIP60* was detectable in all tested tissues, including root, internode, leaf, panicle, and developing seeds at different post-anthesis stages. Higher expression levels were observed in roots and mature leaves, whereas relatively weak transcription was detected in internodes, panicles, and early-stage developing seeds. Notably, the transcript abundance of *OsbZIP60* peaked in seeds at 20 days after flowering (DAF), implying that *OsbZIP60* may participate both in vegetative-stage stress response and in rice reproductive development (Figure 1A).

### 2.2. OsbZIP60 Predominantly Localizes to the Nucleus

To determine the subcellular localization of OsbZIP60 protein, the full coding sequence of *OsbZIP60* (excluding stop codon) was fused to the GFP reporter and transiently expressed in rice leaf epidermal cells. Confocal laser scanning microscopy revealed strong GFP fluorescence signals that fully overlapped with the nuclear marker Ghd7-CFP in the merged channel, confirming that OsbZIP60 predominantly accumulates in the nucleus (Figure 1B).

### 2.3. OsbZIP60 Is Induced by Osmotic, Hormonal, Oxidative and Salt-Stress Signals

To determine whether *OsbZIP60* responds to abiotic stress-related signals, we analyzed its transcript levels under PEG, ABA, H_2_O_2_ and NaCl treatments. Under PEG treatment, *OsbZIP60* expression increased rapidly and reached a strong peak at 6 h, followed by a gradual decrease at 12 h and 24 h. ABA treatment also induced *OsbZIP60*, with strong transcript accumulation at 1 h, 6 h and 24 h. H_2_O_2_ treatment increased *OsbZIP60* expression, with the highest level observed at 12 h. Under NaCl treatment, *OsbZIP60* expression was progressively induced and reached its highest level at 24 h.

**Figure 2:**
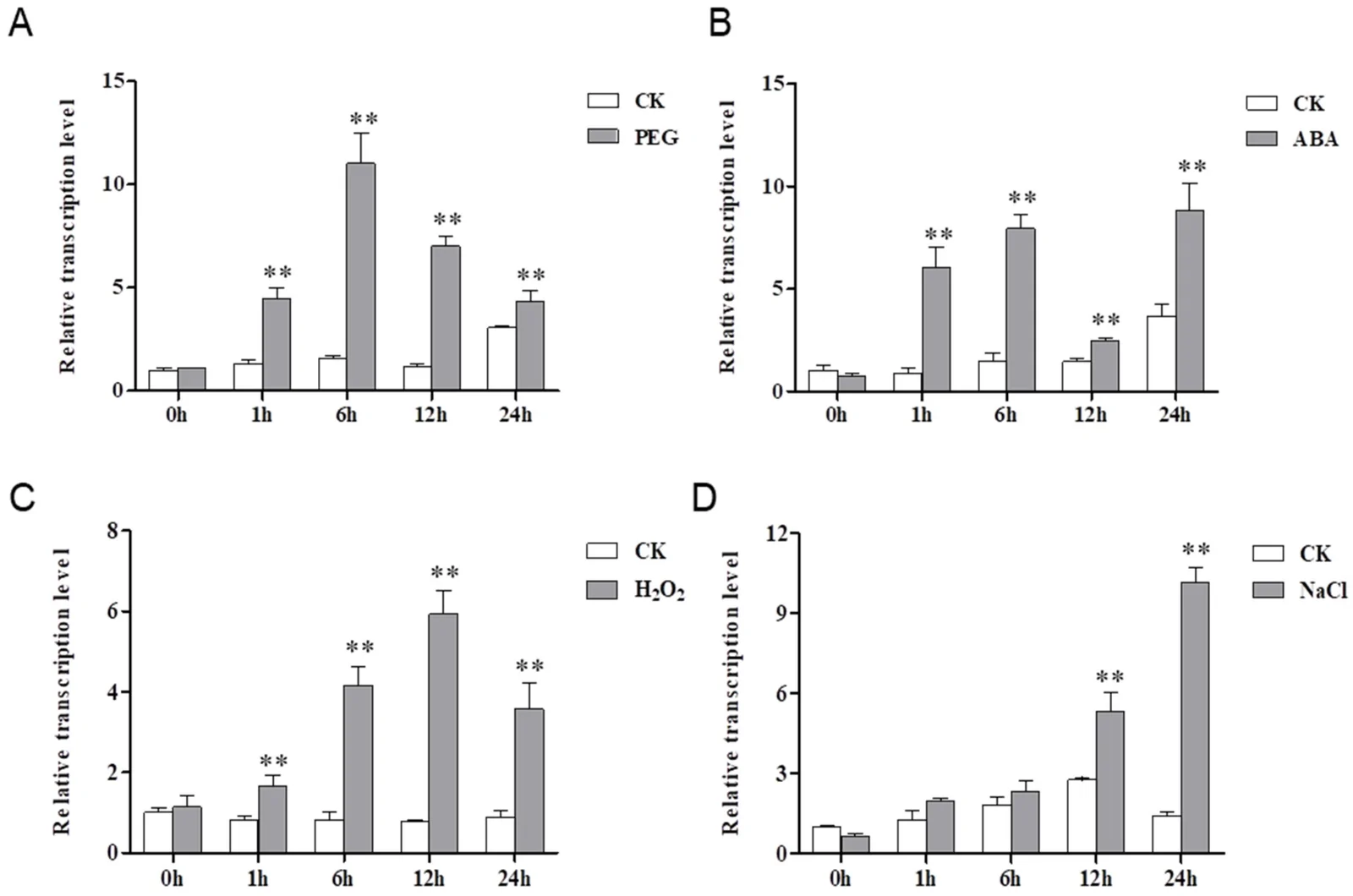
*OsbZIP60* expression is induced by multiple stress-related signals. (**A**–**D**), Relative expression levels of *OsbZIP60* in rice seedlings after treatment with PEG, ABA, H_2_O_2_ and NaCl for 0, 1, 6, 12 and 24 h. CK indicates untreated control plants at the corresponding time points. Error bars denote standard deviation (SD); only upper-side error bars are presented. Significant differences are marked with asterisks (*n* = 3).

These results indicate that *OsbZIP60* is responsive to multiple stress-related signals. The induction by NaCl and PEG suggests involvement in salt and osmotic stress response, whereas induction by ABA and H_2_O_2_ implies that *OsbZIP60* may act in ABA-dependent and ROS-related signaling pathways (Figure 2).

### 2.4. Overexpression of OsbZIP60 Enhances Rice Tolerance to NaCl Stress

**Figure 3:**
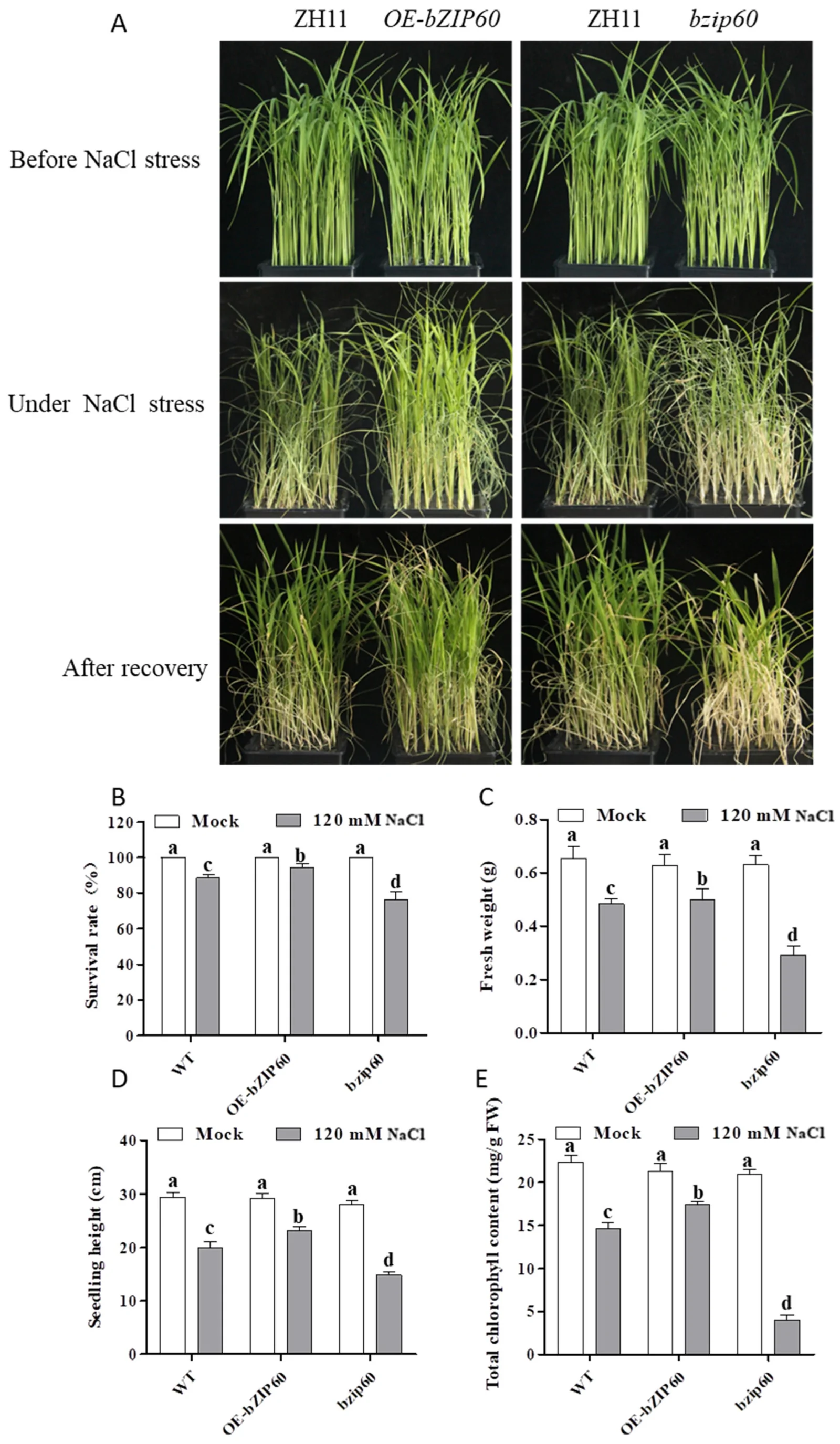
*OsbZIP60* positively regulates salt-stress tolerance in rice seedlings. (**A**) Phenotypic performance of wild-type ZH11, *OE-bZIP60* overexpression lines, and *bzip60* knockout mutants before NaCl stress, under 120 mM NaCl stress, and after recovery culture (note that the photographs of ZH11 at each treatment stage were taken from the same set of individual plants.). (**B**–**E**) Statistical quantification of survival rate, fresh weight, seedling height, and total chlorophyll content of WT, *OE-bZIP60*, and *bzip60* plants under mock and 120 mM NaCl treatments. Error bars represent standard deviation (SD), and only upper-direction error bars are displayed. Different lowercase letters above bars indicate significant differences among genotypes and treatments at *p* < 0.05 (*n* = 12).

To dissect the biological function of *OsbZIP60* in salt-stress tolerance, wild-type Zhonghua 11 (ZH11), *OsbZIP60*-overexpressing (*OE-bZIP60*) transgenic lines, and *bzip60* knockout mutants were subjected to 120 mM NaCl stress treatment. Under normal growth conditions, no obvious phenotypic differences were observed among the three genotypes. After continuous NaCl treatment, *OE-bZIP60* seedlings maintained greener leaves and superior growth status, whereas *bzip60* mutants exhibited severe leaf chlorosis, wilting, and significant growth inhibition. After a seven-day recovery period under normal hydroponic conditions, *OE-bZIP60* plants displayed higher survival rates and better growth vigor, while *bzip60* mutants suffered irreversible severe damage (Figure 3A).

Quantitative physiological measurements further validated the phenotypic observations. Under NaCl stress, the survival rate, fresh biomass, seedling height, and total chlorophyll content of *OE-bZIP60* plants were significantly higher than those of wild-type ZH11. In contrast, *bzip60* mutants exhibited drastically reduced survival ratio, biomass accumulation, seedling height, and chlorophyll concentration. Collectively, phenotypic and physiological differences were observed among wild type, overexpression and knockout genotypes under salt-stress and recovery conditions (Figure 3B–E).

### 2.5. OsbZIP60 Promotes Osmotic Adjustment and Modulates Antioxidant Defense Systems Under Salt-Stress

**Figure 4:**
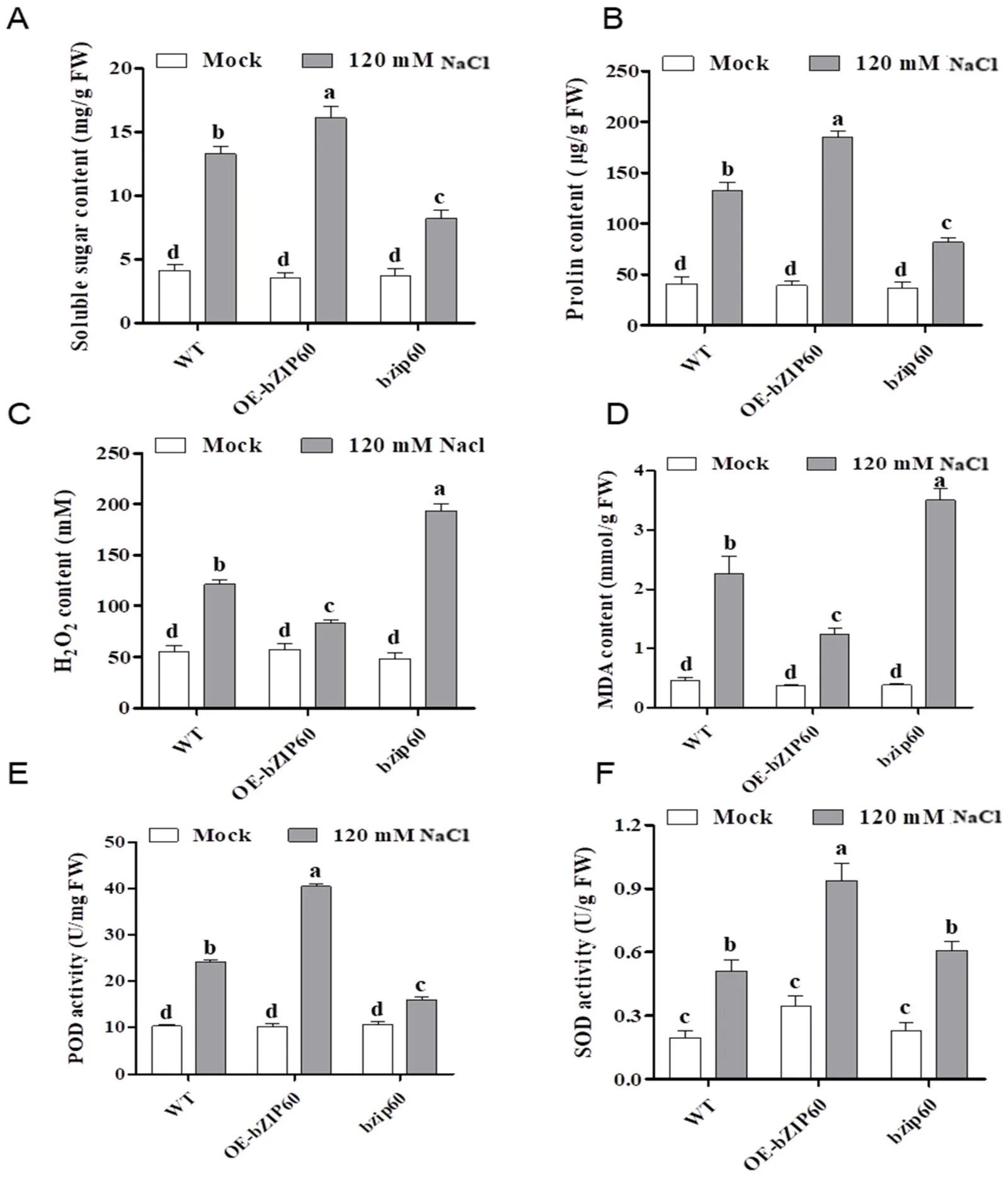
OsbZIP60 enhances osmotic adjustment and antioxidant capacity under salt-stress. Soluble sugar content (**A**), proline content (**B**), H_2_O_2_ content (**C**), MDA content (**D**), POD activity (**E**) and SOD activity (**F**) in WT, *OE-bZIP60* and *bzip60* plants under mock and 120 mM NaCl treatments. Error bars represent standard deviation (SD), and only upper-direction error bars are displayed. Different lowercase letters indicate significant differences at *p* < 0.05 (*n* = 3).

To explore the physiological basis of *OsbZIP60*-mediated salt tolerance, we quantified endogenous osmoprotectant contents and oxidative stress biomarkers across all genotypes. Under NaCl stress, soluble sugar and proline concentrations increased in all tested rice materials, yet the accumulation of both osmoprotectants was significantly higher in *OE-bZIP60* lines compared with wild-type plants. Conversely, *bzip60* mutants accumulated markedly lower levels of soluble sugars and proline (Figure 4A,B). These results indicate that *OsbZIP60* enhances the osmotic adjustment capacity of rice cells under saline conditions.

We next measured H_2_O_2_ and MDA contents to evaluate oxidative stress and membrane lipid peroxidation. Under stress, *bzip60* mutants accumulated markedly higher levels of H_2_O_2_ and MDA than the wild type, whereas *OsbZIP60*-overexpressing plants showed significantly lower H_2_O_2_ and MDA levels (Figure 4C,D). Consistently, antioxidant enzyme activities were enhanced in the overexpression plants. POD and SOD activities were significantly higher in *OsbZIP60*-overexpressing plants but lower in *bzip60* mutants under stress (Figure 4E,F). These physiological changes indicate that OsbZIP60 improves salt tolerance by modulating antioxidant defense.

### 2.6. OsbZIP60 Contributes to Ion Homeostasis Under Stress

**Figure 5:**
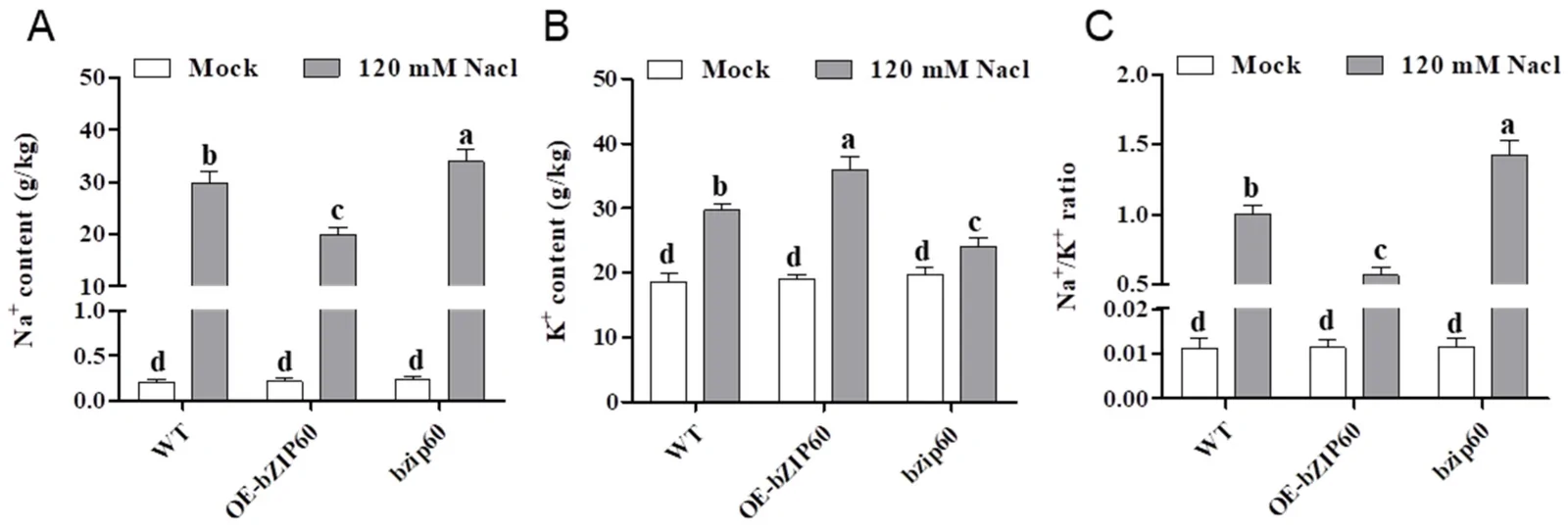
OsbZIP60 improves Na^+^/K^+^ homeostasis under stress conditions. Na^+^ content (**A**), K^+^ content (**B**) and Na^+^/K^+^ ratio (**C**) in WT, *OE-bZIP60* and *bzip60* plants under mock and stress treatment. Error bars represent standard deviation (SD), and only upper-direction error bars are displayed. Different lowercase letters indicate significant differences at *p* < 0.05 (*n* = 3).

Ion homeostasis is a key determinant of rice salt tolerance. To determine whether *OsbZIP60* affects ion balance, Na^+^ and K^+^ contents as well as the Na^+^/K^+^ ratio were analyzed under the stress treatment shown in the dataset. Under stress conditions, Na^+^ content increased markedly in all genotypes, but the increase was lower in *OsbZIP60*-overexpressing plants and higher in *bzip60* mutants than in the wild type (Figure 5A). K^+^ content was better maintained in the overexpression plants, whereas *bzip60* mutants showed lower K^+^ accumulation under stress (Figure 5B). Accordingly, the Na^+^/K^+^ ratio was lowest in the overexpression plants and highest in the mutants (Figure 5C). These results suggest that OsbZIP60 contributes to stress tolerance partly by restricting excessive Na^+^ accumulation and maintaining a favorable Na^+^/K^+^ balance.

### 2.7. OsbZIP60 Modulates Transcript Levels of Salt-Responsive and Antioxidant-Related Genes

**Figure 6:**
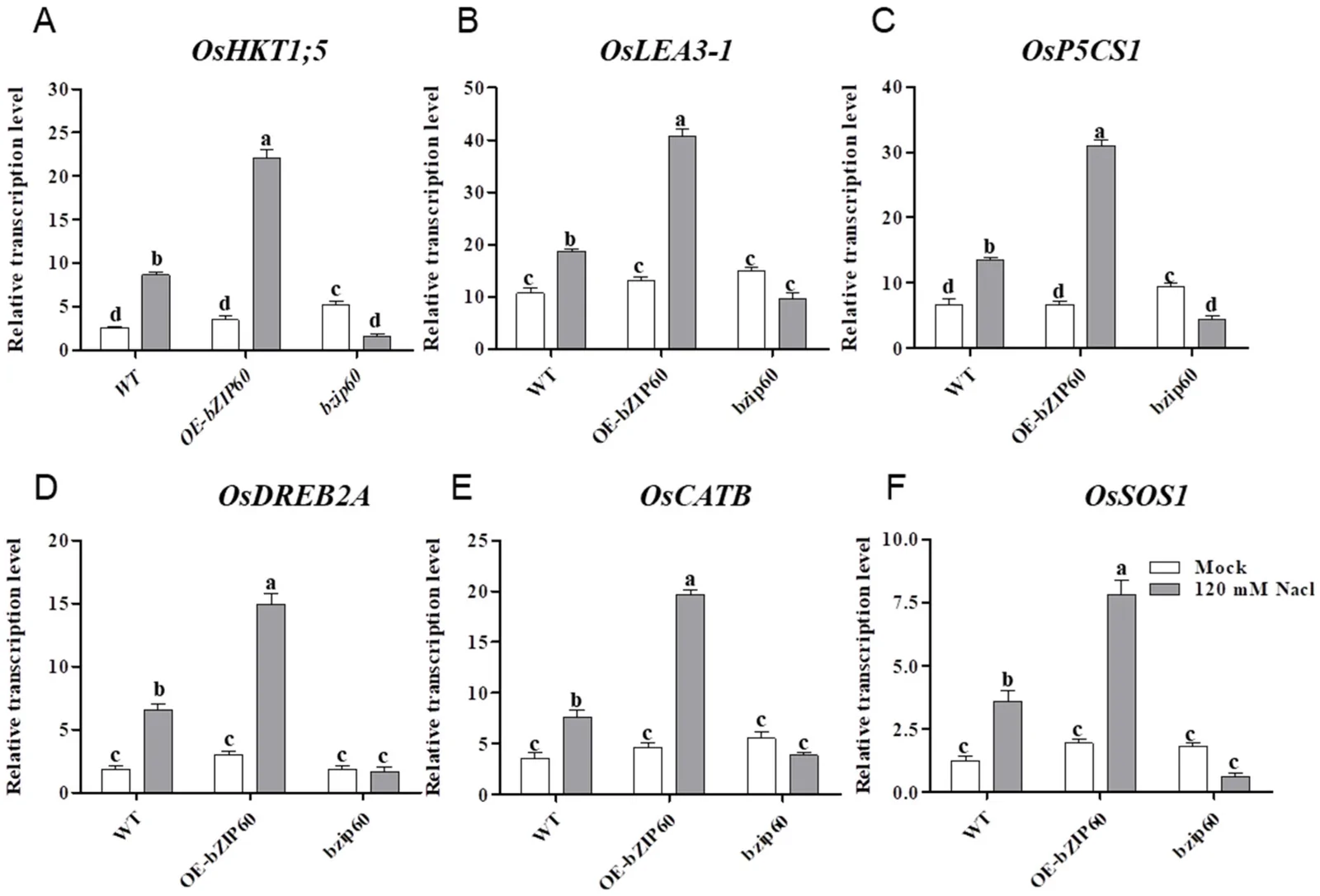
*OsbZIP60* regulates the expression of stress-responsive genes under NaCl stress. Relative transcript levels of *OsHKT1;5* (**A**), *OsLEA3-1* (**B**), *OsP5CS1* (**C**), *OsDREB2A* (**D**), *OsCATB* (**E**) and *OsSOS1* (**F**) in WT, *OE-bZIP60* and *bzip60* plants under mock and 120 mM NaCl treatments. Error bars indicate standard deviation (SD); only upper-side error bars are shown in the charts. Different lowercase letters indicate significant differences at *p* < 0.05 (*n* = 3).

To further dissect the molecular changes associated with OsbZIP60-mediated salt tolerance, we examined the transcript abundance of six well-characterized salt responsive marker genes covering ion homeostasis, osmotic protection, transcriptional regulation, and ROS scavenging pathways in wild type, *OE-bZIP60*, and *bzip60* knockout seedlings under mock and NaCl-treated conditions (Figure 6). The selected candidate genes included *OsHKT1;5* (*LOC_Os01g20160*), *OsLEA31* (*LOC_Os05g46480*), *OsP5CS1* (*LOC_Os05g38150*), *OsDREB2A* (*LOC_Os01g07120*), *OsCATB* (*LOC_Os06g51150*), and *OsSOS1* (*LOC_Os12g44360*). Under salt stress treatment, the transcript levels of all six tested genes were significantly upregulated in *OsbZIP60*-overexpressing plants compared with the wild type control (Figure 6A–F). In contrast, these genes exhibited weaker induction or diminished transcript accumulation in *bzip60* knockout mutants upon salinity exposure.

*OsHKT1;5* and *OsSOS1* function in Na^+^ transport and ion homeostasis; *OsP5CS1* participates in proline biosynthesis for osmotic adjustment; *OsLEA31* confers cellular protection against osmotic dehydration; *OsDREB2A* encodes a stress-responsive transcription factor; and *OsCATB* encodes a catalase isoform responsible for ROS detoxification. Distinct transcript profiles of these stress-associated genes were observed among wild type, *OE-bZIP60*, and *bzip60* genotypes following NaCl challenge (Figure 6A–F).

## 3. Discussion

### 3.1. Stress-Responsive Nuclear Localization of OsbZIP60 and Cross-Species Comparison with bZIP60 Homologs

Members of the bZIP transcription-factor family are widely documented as central participants in plant abiotic-stress responses across numerous plant species. In rice, several well-characterized bZIP proteins, such as OsbZIP23, OsbZIP71 and OsABI5, exert important functions in ABA signaling salt and drought adaptation [7,46,51], yet functional information for OsbZIP60 remains fragmentary compared with these paralogs.

In the present study, our tissue-expression data show that *OsbZIP60* is expressed throughout multiple rice tissues, with relatively high transcript abundance in roots and leaves, and notable accumulation in developing seeds (Figure 1A). Subcellular localization experiments confirm that OsbZIP60 resides in the cell nucleus (Figure 1B), consistent with the subcellular feature of canonical bZIP transcriptional regulators [1]. Time-course expression assays further reveal that *OsbZIP60* transcript levels are responsive to NaCl, PEG-simulated osmotic stress, ABA and H_2_O_2_ treatments (Figure 2).

Homologous bZIP60 proteins have been functionally characterized in only a handful of plant species. In *Arabidopsis thaliana*, AtbZIP60 acts as a core endoplasmic-reticulum (ER)-stress-responsive transcription factor that mediates the unfolded protein response (UPR), yet few studies have documented its contribution to salt or oxidative-stress adaptation [52,53]. In contrast, our expression analyses demonstrate that OsbZIP60 in rice is markedly induced by salt, osmotic, ABA, and oxidative treatments, indicating clear functional divergence between rice *OsbZIP60* and its *Arabidopsis* ortholog. In maize, the UPR-associated bZIP60 transcription factor directly binds to the promoter of *HSFTF13* (an *HSFA6B*-family gene) and regulates transcript levels of heat-shock protein coding and chlorophyll catabolism-related genes, uncovering an unanticipated molecular connection between the ER localized UPR and the nuclear/cytoplasmic heat shock response (HSR) [54]. Such inter-species functional divergence is commonly observed within the bZIP transcription factor family: homologous proteins can evolve divergent stress response outputs even when their protein domains remain conserved. To date, functional investigations of *bZIP60* orthologs in crop plants remain scarce, and further comparative analyses are needed to fully decipher its biological functions across cereal species.

### 3.2. Genetic Evidence for OsbZIP60-Mediated Salt Tolerance in Rice

Phenotypic evaluation under salt-stress demonstrates that overexpression of *OsbZIP60* improves rice salt-stress performance, whereas the *bzip60* knockout mutant displays aggravated salt sensitivity (Figure 3). Physiological measurements further reveal genotype-dependent differences in osmoprotectant accumulation, ROS-related physiological markers, antioxidant enzyme activities, and Na^+^/K^+^ homeostasis under saline conditions (Figure 4 and Figure 5).

Many rice transcription-factor genes confer salt tolerance by modulating multiple physiological processes simultaneously. For instance, *OsMADS27* and the *miR396b-GRF6* module improve salt resistance by coordinating osmolyte accumulation, antioxidant capacity and ion homeostasis [19,27]. Per the phenotypic and physiological observations obtained in our study, OsbZIP60 can be placed into this group of multi-process positive regulators of rice salt acclimation. Based on our experimental data, we can conclude that functional OsbZIP60 is required for full salt-stress adaptation in rice seedlings. However, our physiological assays cannot resolve whether these downstream physiological changes represent direct consequences of OsbZIP60 activity or secondary physiological outputs arising from overall altered-stress status among genotypes.

### 3.3. Genotype Associated Expression of Stress-Related Genes

Salt-stress triggers massive accumulation of reactive oxygen species, which can provoke lipid peroxidation, membrane damage, and impaired cellular metabolism when ROS concentrations exceed homeostatic thresholds. Malondialdehyde (MDA) is widely used as a physiological marker for oxidative membrane injury, while enzymatic ROS-scavenging systems (including SOD and POD) mitigate oxidative damage and sustain redox equilibrium. In the present study, *bzip60* knockout mutants accumulated higher H_2_O_2_ and MDA contents after salt treatment, indicating severe oxidative damage. In contrast, *OsbZIP60*-overexpressing lines maintained lower ROS and MDA levels accompanied by significantly higher SOD and POD enzymatic activities (Figure 4C–F).

At the molecular level, the antioxidant enzyme coding gene *OsCATB* showed stronger salt-induced transcript accumulation in *OsbZIP60*-overexpression lines and diminished induction in *bzip60* knockout mutants (Figure 6E). Similar regulatory patterns for antioxidant-associated genes have been reported for several other rice stress regulators, such as *miR396b-GRF6* and *OsMADS27*, highlighting the importance of antioxidant capacity for salt-stress resilience [19,27]. Considering that *OsbZIP60* itself is transcriptionally induced by H_2_O_2_, we hypothesize a potential feedback loop: salt-stress triggers ROS accumulation that elevates *OsbZIP60* expression; OsbZIP60-dependent physiological changes then reinforce antioxidant capacity to restrict excess ROS buildup. Nevertheless, the molecular hierarchy underlying this putative feedback circuit remains to be biochemically verified.

### 3.4. OsbZIP60-Associated Na^+^/K^+^ Homeostasis Under Salt-Stress

Ionic toxicity constitutes one of the predominant deleterious outcomes occurring at the later phase of salt exposure in rice [55]. Excess apoplastic Na^+^ passively permeates plant cells and competitively inhibits root K^+^ acquisition. Disrupted cellular Na^+^/K^+^ stoichiometry impairs enzymatic function, perturbs core cellular metabolism, and compromises plasma membrane integrity [56]. Accordingly, sustaining low cytosolic Na^+^ concentrations, sufficient K^+^ retention, and a balanced Na^+^/K^+^ ratio is widely recognized as a hallmark physiological trait of salt-tolerant rice genotypes. Consistent with this established physiological framework, our ion-profiling measurements uncovered genotype dependent alterations in ionic homeostasis upon salinity challenge. Compared with wild type seedlings, *OsbZIP60*-overexpressing plants maintained reduced Na^+^ accumulation and superior K^+^ retention, yielding a lowered cellular Na^+^/K^+^ ratio, whereas *bzip60* knockout mutants exhibited severely perturbed Na^+^-K^+^ balance (Figure 5). These observations establish a clear genetic linkage between functional OsbZIP60 and the preservation of ionic homeostasis during salt stress.

Two pivotal sodium transport encoding genes, *OsHKT1;5* (*SKC1*) and *OsSOS1*, displayed genotype correlated transcript shifts under salt treatment [57]. *OsHKT1;5* mediates Na^+^ retrieval from xylem sap to mitigate toxic Na^+^ delivery to photosynthetic leaves, while *OsSOS1* encodes a plasma membrane localized Na^+^/H^+^ antiporter that extrudes Na^+^ out of root epidermal cells; together they cooperatively alleviate Na^+^ derived cytotoxicity in rice shoots. Nevertheless, these observed transcriptional alterations of *OsHKT1;5* and *OsSOS1* cannot be interpreted as definitive evidence for OsbZIP60-dependent direct transcriptional regulation. As elaborated in Section 3.3, altered transcript abundance within transgenic and mutant backgrounds may stem from broad-scale physiological reprogramming triggered by salinity, rather than locus-specific transcriptional control. Hence, it remains unresolved whether OsbZIP60 modulates ion homeostasis by directly activating the transcription of these two transporter genes, or via indirect downstream physiological cascades.

Resolving this mechanistic ambiguity demands orthogonal biochemical validation assays [27]. Combined in vitro and in vivo protein–DNA interaction assays, including yeast one-hybrid screening, ChIP-seq, and EMSA, are required to interrogate physical interactions between OsbZIP60 and bZIP type *cis*-regulatory motifs harbored within the promoters of *OsHKT1;5* and *OsSOS1*. Such follow-up biochemical investigations will help dissect the precise molecular routes through which OsbZIP60 participates in the regulatory module governing salt-triggered ion homeostasis adjustment in rice.

## 4. Materials and Methods

### 4.1. Plant Materials and Growth Conditions

Rice (*Oryza sativa* L. ssp. *japonica*) cultivar Zhonghua 11 (ZH11) served as wild-type control. T4 generation *OsbZIP60*-overexpressing lines and T5 generation *bzip60* knockout mutants were obtained from another laboratory. All plants used for phenotyping were genotyped to verify the corresponding transgenic or knockout alleles. Comprehensive molecular characterization (transgene copy-number and insertion-site analysis) of these materials was previously reported and not repeated in this study [58].

After surface sterilization, seeds were germinated and hydroponically cultured under controlled growth chamber conditions (32 °C light/28 °C dark) in nutrient solution containing 1.15 mM (NH_4_)_2_SO_4_, 0.2 mM NaH_2_PO_4_, 1 mM CaCl_2_, 1 mM MgSO_4_, 0.4 mM K_2_SO_4_, 9 μM MnCl_2_, 19 μM H_3_BO_3_, 0.152 μM ZnSO_4_, 0.155 μM CuSO_4_, 0.075 μM (NH_4_)_6_Mo_7_O_24_, and 20 μM Fe-EDTA (pH 5.8). All experimental treatments with PEG, ABA, H_2_O_2_ and NaCl were performed referring to previous studies [59,60,61]. Briefly, two-week-old rice seedlings were subjected to 15% (*w*/*v*) PEG6000, 100 μM ABA, 5 mM H_2_O_2_, and 120 mM NaCl, respectively, for stress treatments. The 14-day-old seedlings of WT and transgenic lines were treated with 120 mM NaCl for one week. Seedlings grown in nutrient solution without NaCl served as controls. After treatment, the survival rate, fresh weight, seedling height, and total chlorophyll content were measured. Each treatment experiment was performed with three replicates with twelve seedlings.

### 4.2. Chlorophyll Content Measurement

For quantification of total chlorophyll content, approximately 0.1 g fresh leaf tissues from both treated and control rice plants were cut into small fragments and immersed in 10 mL of ethanol-acetone mixed solution (1:1, *v*/*v*). Pigment extraction was performed in complete darkness for 48 h until leaf tissues turned fully bleached. The absorbance of extracts was determined at 663 nm and 645 nm using a UV-visible spectrophotometer. Three independent biological replicates were included for each sample. Chlorophyll concentrations were calculated following previously published equations [62].

### 4.3. Quantitative Reverse Transcription (qRT)-PCR

Total RNA was isolated using an RNA extraction kit supplied by YESEN Biotech (Shanghai, China). First-strand complementary DNA (cDNA) was reverse-transcribed with PrimeScript RT Master Mix (Takara, Dalian, China). Quantitative real-time PCR (qRT-PCR) for target genes was subsequently run on a CFX96 real-time thermal cycler manufactured by Bio-Rad (California, USA). All samples were analyzed in triplicate technical replicates. The ubiquitin gene *UBQ* (*LOC_Os03g13170*) served as the reference gene for normalization. Relative transcript abundance was quantified via the 2^−ΔΔCT^ algorithm. All primer sequences exploited throughout this work are summarized in Supplementary Table S1.

### 4.4. Subcellular Localization

The coding sequence of *OsbZIP60* without the stop codon was amplified and fused with pCA1301-35S-S65T-GFP. The *OsbZIP60*-GFP construct and Ghd7-CFP marker were transiently expressed in plant epidermal cells. Fluorescence signals were observed using confocal laser scanning microscopy (Karl Zeiss LSM710, Jena, Germany).

### 4.5. Stress Treatments

For gene-expression analysis under abiotic stress, rice seedlings hydroponically cultured in nutrient solution described in Section 4.1 (pH 5.8) were subjected to different stress treatments at the three-leaf stage. For salt-stress, 120 mM NaCl was supplemented into the nutrient solution. For osmotic stress, 15% (*w*/*v*) PEG-6000 was added to the culture medium. For phytohormone and oxidative-stress treatments, seedlings were exposed to 100 μM ABA and 5 mM H_2_O_2_, respectively, by adding corresponding stock solutions to the hydroponic medium. Seedlings grown in normal nutrient solution without additional stress agents served as the untreated control. Leaf tissues were harvested at 0, 1, 6, 12 and 24 h post-treatment. Total RNA was isolated from collected samples, and transcript abundance of *OsbZIP60* was quantified via qRT-PCR.

### 4.6. Physiological Measurements

The soluble sugar concentration was quantified using the anthrone-sulfuric acid colorimetric method. Briefly, leaf samples were ground with 80% ethanol solution in a water-bath for extraction. After centrifugation, the supernatant was mixed with anthrone reagent, followed by boiling incubation. The absorbance of the reaction mixture was measured at 620 nm, and soluble sugar contents were calculated referring to the glucose standard curve.

Free proline accumulation was assayed via sulfosalicylic-acid extraction combined with ninhydrin chromogenic reaction as previously described [63]. Leaf homogenate was mixed with the chromogenic mixture, incubated at 100 °C for 15 min, and extracted with toluene after cooling. The absorbance of the toluene phase was recorded at 520 nm to calculate proline levels.

Endogenous H_2_O_2_ and MDA contents were determined by thiobarbituric acid (TBA) colorimetry. Briefly, leaf powder was homogenized in 10% trichloroacetic acid (TCA) containing 0.25% TBA. After centrifugation, the supernatant was heated in a 95 °C water bath, cooled and subjected to spectrophotometric detection at 450 nm, 532 nm and 600 nm for the calculation of H_2_O_2_ and MDA concentrations.

For antioxidant enzyme activity analysis, leaf samples were fully pulverized in liquid nitrogen and homogenized with pre-cooled phosphate buffer (pH 7.2) to obtain crude enzyme extract. POD activity was determined by monitoring the oxidation rate of guaiacol at 470 nm, with one unit of enzyme activity defined as the absorbance change of 0.01 per minute. SOD activity was assayed based on the inhibitory effect on pyrogallol auto-oxidation at 320 nm; one SOD unit represented the enzyme amount required to suppress auto-oxidation by 50%.

### 4.7. Ion Content Analysis

Shoot tissues were sampled from wild-type (WT) and transgenic rice seedlings under both normal growth and salt-stress conditions to quantify endogenous cation levels. Collected plant materials underwent a two-stage drying procedure: samples were first deactivated at 105 °C for 30 min, then continuously dried at 75 °C over 48 h to achieve constant weight. The subsequent quantification of Na^+^ and K^+^ levels was implemented following the well-established analytical protocol published in a prior study [63].

## Data Availability

All data generated or analyzed during this study are included in this published article.

## Supplementary Materials

The following supporting information can be downloaded at: https://www.mdpi.com/article/10.3390/ijms27188143/s1.

## References

[1] Guo P., Chong L., Jiao Z., Xu R., Niu Q., Zhu Y. (2025). Salt stress activates the CDK8-AHL10-SUVH2/9 module to dynamically regulate salt tolerance in Arabidopsis. Nat. Commun..

[2] Zhang Y., Liu S., Liang X., Zheng J., Lu X., Zhao J., Li H., Zhan Y., Teng W., Li H. (2025). GmFER1, a soybean ferritin, enhances tolerance to salt stress and root rot disease and improves soybean yield. Plant Biotechnol. J..

[3] Zhou H., Shi H., Yang Y., Feng X., Chen X., Xiao F., Lin H., Guo Y. (2024). Insights into plant salt stress signaling and tolerance. J. Genet. Genom. Yi Chuan Xue Bao.

[4] Colin L., Ruhnow F., Zhu J.K., Zhao C., Zhao Y., Persson S. (2023). The cell biology of primary cell walls during salt stress. Plant Cell.

[5] Liu Z., Ma C., Hou L., Wu X., Wang D., Zhang L., Liu P. (2022). Exogenous SA affects rice seed germination under salt stress by regulating Na^+^/K^+^ balance and endogenous GAs and ABA homeostasis. Int. J. Mol. Sci..

[6] Duan S., Zhao L., Jiang R., Deng R., Zhou H., Mohammed Y.Z., Yue N., Yang X., Zhang M., Wang B. (2026). Drill seeding and 5-ALA improve photosynthetic efficiency and ion balance of rice seedlings under salt stress. BMC Plant Biol..

[7] Li Y., Zhou J., Li Z., Qiao J., Quan R., Wang J., Huang R., Qin H. (2022). SALT AND ABA RESPONSE ERF1 improves seed germination and salt tolerance by repressing ABA signaling in rice. Plant Physiol..

[8] Fernández-Ramírez V.J., De la Rubia A.G., Pardo J.M., Quintero F.J., Gámez-Arjona F.M. (2026). The architecture of salt tolerance: A multi-scale view of sodium transport in plants. Plant Commun..

[9] Zhang Z., Wei J., Wei Y., Yan M., Guo J., Jiang X., Wei Z., Liu Y., Zhang D., Yang C. (2026). The MdERF1B-MdWRKY75-MdSOS3 module confers salt tolerance by regulating sodium-potassium homoeostasis in apple. Plant Cell Environ..

[10] Liu W., Zhang Z., Du Q., Li J., Yang L., Ren Z., Guo J., Zhu W., Ma Z., Zhou Y. (2026). H3K27me3-mediated chromatin remodeling governs salt tolerance in cotton via Na^+^/K^+^ homeostasis modulation. J. Adv. Res..

[11] Li O., Zou M., Hou X., Zhao J., Zhang H., He C., Fan X., Jin Y., Ma Y., Jia D. (2026). BnaGRP3 mediates salt tolerance via Na(+)/K(+) homeostasis and BnaPIPs interactions in Brassica napus. J. Adv. Res..

[12] Zhu Q., Miao Y., Wang Y., Zhang Z., Ren Z., Zhai X., Li L., Wei S., Li W., Li T. (2026). GhCHX6A and GhKEA1D Enhance Salt Tolerance by Regulating Na^+^/K^+^ Homeostasis in Cotton. Physiol. Plant..

[13] Li C., Guan J., Liang W.H., Yao S., He L., Wei X.D., Zhao L., Zhou L.H., Zhao C.F., Zhao Q.Y. (2025). OsWRKY72 enhances salt tolerance in rice via SKC1-mediated Na+ regulation. Plant Stress.

[14] Wang J., Nan N., Li N., Liu Y., Wang T.J., Hwang I., Liu B., Xu Z.Y. (2020). A DNA Methylation Reader-Chaperone Regulator-Transcription Factor Complex Activates OsHKT1;5 Expression during Salinity Stress. Plant Cell.

[15] Dong N.Q., Lin H.X. (2024). An abundant valuable resource for salt-tolerance allele hunting in rice. Plant Commun..

[16] Yu J., Zhu C., Xuan W., An H., Tian Y., Wang B., Chi W., Chen G., Ge Y., Li J. (2023). Genome-wide association studies identify OsWRKY53 as a key regulator of salt tolerance in rice. Nat. Commun..

[17] Deng P., Jing W., Cao C., Sun M., Chi W., Zhao S., Dai J., Shi X., Wu Q., Zhang B. (2022). Transcriptional repressor RST1 controls salt tolerance and grain yield in rice by regulating gene expression of asparagine synthetase. Proc. Natl. Acad. Sci. USA.

[18] Prakash N.R., Lokeshkumar B.M., Rathor S., Warraich A.S., Yadav S., Vinaykumar N.M., Dushynthkumar B.M., Krishnamurthy S.L., Sharma P.C. (2022). Meta-analysis and validation of genomic loci governing seedling and reproductive stage salinity tolerance in rice. Physiol. Plant..

[19] Alfatih A., Zhang J., Song Y., Jan S.U., Zhang Z.S., Xia J.Q., Zhang Z.Y., Nazish T., Wu J., Zhao P.X. (2023). Nitrate-responsive OsMADS27 promotes salt tolerance in rice. Plant Commun..

[20] Liu Y., Zhu Q., Li R., Wei R., Geng X., Zhang X., Wei H., Gao P., Xu K., Dai Q. (2025). Foliar magnesium enhances rice salt tolerance by improving photosynthesis and regulating ion homeostasis. Front. Plant Sci..

[21] An J., Qian Y., Xie Z., Wang W., Chen K., Zhang G., Jiang J., Xu J., Zheng T., Zhang F. (2026). Lhca4 maintaining photochemical efficiency and ROS homeostasis contributes to rice salt tolerance. Rice.

[22] Melnikava A., Perreau F., Paniagua C., Cheng S.C., Huang L.M., Pekarova B., Berendzen K.W., Mira-Rodado V., Citterico M., Tvrdonova A. (2026). Cytokinin-activated DIRIGENT13 promotes lignan synthesis to regulate root growth and abiotic stress tolerance by modulating ROS homeostasis in Arabidopsis. Plant Commun..

[23] Zhu Z., Wang J., Liu H., Zhang Y., Zhao X., Wang C. (2026). ThASR4 enhances salt tolerance in transgenic Tamarix and Arabidopsis by scavenging reactive oxygen species. BMC Plant Biol..

[24] Liu F., Huang X., Li X., Yu M., Wegner L.H. (2026). Salt tolerance in the rice varieties Minghui63 and Nipponbare: Root growth reduction correlates with local ROS accumulation and net H^+^ flux patterns at the root apex. J. Plant Physiol..

[25] Shang Y., Tian Z., Zhang Y., Song S., Guo Y., Song Z., Mo H., Wang K., Zhao Y., Zhang H. (2026). Overexpression of PagAPX8 promotes poplar growth and enhances tolerance to drought and salt stress. Plant Cell Rep..

[26] Mou H.B., Jiao S., Zheng X.M., Liu X.H., Li Z., Xun J.N., Zhao K., Feng X., Zhai H., Bai X. (2026). The soybean gene GmSP1L-8 improves salt tolerance through the enhancement of the ROS scavenging system. Plant Sci..

[27] Yuan H., Cheng M., Wang R., Wang Z., Fan F., Wang W., Si F., Gao F., Li S. (2024). miR396b/GRF6 module contributes to salt tolerance in rice. Plant Biotechnol. J..

[28] Bigham Soostani S., Ranjbar M., Memarian A., Mohammadi M., Yaghini Z. (2025). Regulation of APX, SOD, and PAL genes by chitosan under salt stress in rapeseed (*Brassica napus* L.). BMC Plant Biol..

[29] Nie S., Huang W., He C., Wu B., Duan H., Ruan J., Zhao Q., Fang Z. (2025). Transcription factor OsMYB2 triggers amino acid transporter OsANT1 expression to regulate rice growth and salt tolerance. Plant Physiol..

[30] Zuo G., Mei W., Feng N., Zheng D. (2025). Photosynthetic performance index (PIabs) and malondialdehyde (MDA) content determine rice biomass under combined salt stress and prohexadione-calcium treatment. BMC Plant Biol..

[31] Luo C., Akhtar M., Min W., Alam Y., Ma T., Shi Y., She Y., Lu X. (2023). The suppressed expression of a stress responsive gene ‘OsDSR2’ enhances rice tolerance in drought and salt stress. J. Plant Physiol..

[32] Yang L., Fang S., Liu L., Zhao L., Chen W., Li X., Xu Z., Chen S., Wang H., Yu D. (2025). WRKY transcription factors: Hubs for regulating plant growth and stress responses. J. Integr. Plant Biol..

[33] Zhang D., Zhou H., Zhang Y., Zhao Y., Zhang Y., Feng X., Lin H. (2025). Diverse roles of MYB transcription factors in plants. J. Integr. Plant Biol..

[34] Feng Y., Xia P. (2025). Heat shock transcription factors as integrative hubs for plant stress adaptation: Decoding regulatory networks toward climate-resilient crop design. Plant Cell Environ..

[35] Liu X., Guo X., Li T., Wang X., Guan Y., Wang D., Wang Y., Ji X., Gao Q., Ji J. (2025). OsGSK1 interacts with OsbZIP72 to regulate salt response in rice. Plant J..

[36] Gao Y., Zhao X., Liu X., Liu C., Zhang K., Zhang X., Zhou J., Dong G., Wang Y., Huang J. (2024). OsRAV1 regulates seed vigor and salt tolerance during germination in rice. Rice.

[37] Kim Y., Kim B., Kang J., Bae S.I., Yoon H., Shin H.J., Lee J.Y., Paek N.C., Kang K. (2025). ONAC005 enhances salt stress tolerance by promoting suberin deposition in root endodermis. Plant J..

[38] Xiao L., Shi Y., Wang R., Feng Y., Wang L., Zhang H., Shi X., Jing G., Deng P., Song T. (2022). The transcription factor OsMYBc and an E3 ligase regulate expression of a K+ transporter during salt stress. Plant Physiol..

[39] Peng L., Chao D., Sun X., Li L., Yu J., Li Z., Zhang B., Liu C., Fu S., Chen Z. (2025). OsWRKY18, a WRKY transcription factor, is involved in rice salt tolerance. Plant Cell Physiol..

[40] Wu J., Yu C., Huang L., Gan Y. (2021). A rice transcription factor, OsMADS57, positively regulates high salinity tolerance in transgenic Arabidopsis thaliana and Oryza sativa plants. Physiol. Plant..

[41] Luo X., Lu J., Jiang Q., Xu D., Liu Y., Zhang X., Han Y. (2026). Comprehensive analysis for GRF and GIF genes and their responses to abiotic stresses in tea plant. Plant Physiol. Biochem..

[42] Wang T., Li X.K., Liu X., Yang X.Q., Li Y.J., Hou B.K. (2023). Rice glycosyltransferase gene UGT2 functions in salt stress tolerance under the regulation of bZIP23 transcription factor. Plant Cell Rep..

[43] Li J., Tang Y., Li M., Li Y., Shi G., Chen S., Wang Y., Ma Y., Yang C. (2026). OsFLZ5 enhances drought tolerance and ABA sensitivity in rice via transcriptional activation by OsbZIP23. J. Exp. Bot..

[44] Wang Y., Li C., Li X., Yin J., Wang L., Liu Q., Wang Z., Wu Z. (2026). OsDi19-1 acts downstream of OsABI5 to negatively regulate aba-inhibited seed germination and seedling growth in rice. Rice.

[45] Li R., Song Y., Wang X., Zheng C., Liu B., Zhang H., Ke J., Wu X., Wu L., Yang R. (2024). OsNAC5 orchestrates OsABI5 to fine-tune cold tolerance in rice. J. Integr. Plant Biol..

[46] Liu C., Mao B., Ou S., Wang W., Liu L., Wu Y., Chu C., Wang X. (2014). OsbZIP71, a bZIP transcription factor, confers salinity and drought tolerance in rice. Plant Mol. Biol..

[47] Iwata Y., Fedoroff N.V., Koizumi N. (2008). Arabidopsis bZIP60 is a proteolysis-activated transcription factor involved in the endoplasmic reticulum stress response. Plant Cell.

[48] Blanchard C., Aimé S., Ducloy A., Hichami S., Azzopardi M., Cacas J.L., Lamotte O. (2025). The IRE1-bZIP60 branch of the unfolded protein response is required for the Arabidopsis immune response to Botrytis cinerea. J. Exp. Bot..

[49] Xu Z., Zeng T., Li J., Zhou L., Li J., Luo J., Zheng R., Wang Y., Hu H., Wang C. (2023). TcbZIP60 positively regulates pyrethrins biosynthesis in Tanacetum cinerariifolium. Front. Plant Sci..

[50] Yu X., Niu X.L., Yang S.H., Li Y.X., Liu L.L., Tang W., Liu Y.S. (2011). Research on heat and drought tolerance in rice (*Oryza sativa* L.) by Overexpressing Transcription Factor OsbZIP60. Sci. Agric. Sin..

[51] Zong W., Yang J., Fu J., Xiong L. (2020). Synergistic regulation of drought-responsive genes by transcription factor OsbZIP23 and histone modification in rice. J. Integr. Plant Biol..

[52] Tang L., Li G., Wang H., Zhao J., Li Z., Liu X., Shu Y., Liu W., Wang S., Huang J. (2024). Exogenous abscisic acid represses rice flowering via SAPK8-ABF1-Ehd1/Ehd2 pathway. J. Adv. Res..

[53] Sun Q., Duan E., Jing R., Ren Y., Xu H., Gu C., Lv W., Jiang X., Chen R., Wang Q. (2025). The bZIP transcription factor RISBZ1 balances grain filling and ER stress response in rice grains. Plant Commun..

[54] Tang W., Page M. (2013). Transcription factor AtbZIP60 regulates expression of Ca^2+^ -dependent protein kinase genes in transgenic cells. Mol. Biol. Rep..

[55] Li Z., Tang J., Srivastava R., Bassham D.C., Howell S.H. (2020). The transcription factor bzip60 links the unfolded protein response to the heat stress response in maize. Plant Cell.

[56] Yan G., Fan X., Peng M., Yin C., Xiao Z., Liang Y. (2020). Silicon Improves Rice Salinity Resistance by Alleviating Ionic Toxicity and Osmotic Constraint in an Organ-Specific Pattern. Front. Plant Sci..

[57] Wang X., Liu X., Su Y., Shen H. (2025). Rice responses to abiotic stress: Key proteins and molecular mechanisms. Int. J. Mol. Sci..

[58] Jung Y.J., Kim J.Y., Kim H.S., Go J., Kim S.H., Baek J., Kang K.K. (2026). OASA1D-mediated tryptophan enrichment improves redox and ionic homeostasis under salt stress in rice. Int. J. Mol. Sci..

[59] Cao R., Zhao S., Jiao G., Duan Y., Ma L., Dong N., Lu F., Zhu M., Shao G., Hu S. (2022). OPAQUE3, encoding a transmembrane bZIP transcription factor, regulates endosperm storage protein and starch biosynthesis in rice. Plant Commun..

[60] Zhang X., Long Y., Chen X., Zhang B., Xin Y., Li L., Cao S., Liu F., Wang Z., Huang H. (2021). A NAC transcription factor OsNAC3 positively regulates ABA response and salt tolerance in rice. BMC Plant Biol..

[61] Fang Y., Liao K., Du H., Xu Y., Song H., Li X., Xiong L. (2015). A stress-responsive NAC transcription factor SNAC3 confers heat and drought tolerance through modulation of reactive oxygen species in rice. J. Exp. Bot..

[62] Chang Y., Fang Y., Liu J., Ye T., Li X., Tu H., Ye Y., Wang Y., Xiong L. (2024). Stress-induced nuclear translocation of ONAC023 improves drought and heat tolerance through multiple processes in rice. Nat. Commun..

[63] Zhao J., Meng X., Zhang Z., Wang M., Nie F., Liu Q. (2023). OsLPR5 encoding ferroxidase positively regulates the tolerance to salt stress in rice. Int. J. Mol. Sci..

