# Supplementary material for: OsbZIP60 Positively Regulates Salt-Stress Tolerance in Rice
Source: Int J Mol Sci. 2026 Sep 12;27(18):8143. doi: 10.3390/ijms27188143 (PMC13607193; doi:10.3390/ijms27188143)
Supplement: Supplementary file 1 [file ijms-27-08143-s001.zip › ijms-4501241-supplementary.pdf]

Table S1. Primer sequences

Primer	Sequence (5'->3')
qRT-OsbZIP60 F	CCGAGGAATGGGAAGCATGT
qRT-OsbZIP60 R	TTTCCAGAACCCTTCTCGCC
qRT-OsHKT1;5 F	CACCCATTCTGGCTCCAACT
qRT-OsHKT1;5 R	ACCATGCTGGTCTTCATCGG
qRT-OsLEA3-1 F	AGTGAGCAGGTGAAGAGCAC
qRT-OsLEA3-1 R	TGGCAGAGGTGTCCTTGTTG
qRT-OsP5CS1-1 F	CTGTGCGAGCAGGTTAAGGA
qRT-OsP5CS1-1 R	CCGGTACCTAAGTCGCTGTC
qRT-OsDREB2A-1 F	AAGCGATGGCCCTGATTCAA
qRT-OsDREB2A-1 R	CTTTCTTGGACCCCTTGGCT
qRT-OsCATB-1 F	ACACATGAACTCCGTGGCAT
qRT-OsCATB-1 R	TCAGGTTGAGACGTGAAGCC
qRT-OsSOS1-1 F	ATCAGGTGGAGGCTAGAGCA
qRT-OsSOS1-1 R	TGACGCACTCCTTTGCAGAT
